# Surfactant-associated bacteria in the near-surface layer of the ocean

**DOI:** 10.1038/srep19123

**Published:** 2016-01-12

**Authors:** Naoko Kurata, Kate Vella, Bryan Hamilton, Mahmood Shivji, Alexander Soloviev, Silvia Matt, Aurélien Tartar, William Perrie

**Affiliations:** 1 Halmos College of Natural Sciences and Oceanography (formerly Oceanographic Center), Nova Southeastern University, Dania Beach, FL, USA; 2 Rosenstiel School of Marine and Atmospheric Science, University of Miami, Miami, FL, USA; 3 Naval Research Laboratory, Stennis Space Center, MS, USA; 4 Department of Biological Sciences (formerly Division of Math, Science and Technology), Nova Southeastern University, Fort Lauderdale, FL, USA; 5 Fisheries and Oceans Canada, Bedford Institute of Oceanography, Nova Scotia, Canada

## Abstract

Certain marine bacteria found in the near-surface layer of the ocean are expected to play important roles in the production and decay of surface active materials; however, the details of these processes are still unclear. Here we provide evidence supporting connection between the presence of surfactant-associated bacteria in the near-surface layer of the ocean, slicks on the sea surface, and a distinctive feature in the synthetic aperture radar (SAR) imagery of the sea surface. From DNA analyses of the *in situ* samples using pyrosequencing technology, we found the highest abundance of surfactant-associated bacterial taxa in the near-surface layer below the slick. Our study suggests that production of surfactants by marine bacteria takes place in the organic-rich areas of the water column. Produced surfactants can then be transported to the sea surface and form slicks when certain physical conditions are met. This finding has potential applications in monitoring organic materials in the water column using remote sensing techniques. Identifying a connection between marine bacteria and production of natural surfactants may provide a better understanding of the global picture of biophysical processes at the boundary between the ocean and atmosphere, air-sea exchange of greenhouse gases, and production of climate-active marine aerosols.

Bacterial taxa found in thin near-surface layers of the ocean – bacterioneuston^1^,^2^ – are of interest due to a number of practical applications, including air-sea gas exchange of greenhouse gases^3^, production of climate-active marine aerosols^4^ and remote sensing of the ocean. In particular, surfactant (surface active materials) production and degradation via microbial biochemical processes have potential importance in those applications and therefore are a focus of this study.

Known major sources of surfactants in the open ocean include inputs from terrestrial runoff, deposition from atmosphere, and phytoplankton^5^; however, the microbial surfactant production within the ocean has not been fully understood. Microorganisms are important surfactant producers and have received considerable attention from the biochemical industries for the production of detergents, emulsifiers, and dispersants^6^.

Some surfactant-producing marine bacterial strains have been recovered from oil contaminated sites^7^, seawater and sediment^8^,^9^,^10^. Surfactant-producing marine bacteria have wide taxonomic distributions encompassing phyla including *Acinetobacter* spp.*, Arthrobacter* spp.*, Pseudomonas* spp.*, Halomonas* spp.*, Bacillus* spp.*, Rhodococcus* spp., and *Enterobacter* spp^11^,^12^,^13^, which are known to produce variety of surfactants. A standard way to identify surfactant-associated bacteria is to first culture for isolation on medium and to apply various screening methods including drop collapse, oil spreading^14^, hemolytic assay, tilted glass slide, blue agar plate, hydrocarbon overlaid agar plate, emulsification index, and emulsification assay^15^. The conventional surfactant screening methods, however, are highly selective, since environmental marine strains consist of highly complex microbial mixtures and a large number of marine bacterial are difficult to cultivate on medium^16^. Therefore, the culture-dependent methods have limited our understanding of true diversity for surfactant-associated bacteria. However, with the advent of high-throughput sequencing, which revealed an unprecedented number of bacteria present in the ocean, we can argue that there are undiscovered roles that bacteria play in terms of surfactant production and transformation in the oceanic environment.

Surfactants can modify physical properties of the air-sea interface and near-surface layer of the ocean by altering surface tension forces^17^. The effect of surfactants includes dampening of short gravity-capillary waves^18^, as well as the suppression of near-surface turbulence and coherent structures^19^. The presence of surfactants increases the temperature difference across the millimeter thick aqueous thermal molecular diffusion sublayer and the resistance of the air-sea interface to interfacial gas exchange^20^,^21^. Under low and moderate wind speed conditions, surfactants produce slicks (films) on the sea surface, which dampen short gravity-capillary waves^17^. These slicks are also identifiable from space^18^ with high-resolution remote sensing techniques such as synthetic aperture radar (SAR), which responds to short surface (Bragg^22^) scattering waves (Fig. 1).

The top few millimeters of the ocean, where physical, chemical, and biological properties are most altered relative to deeper water, are often referred to as the sea surface microlayer^4^,^23^,^24^. The sea surface microlayer is characterized by the presence of the aqueous viscous, thermal, and diffusion molecular sublayers with extremely large gradients in current velocity, temperature, and gas concentration^23^, respectively. For example, for a typical thickness of 10^−3 ^m and temperature differences across the thermal molecular diffusion sublayer of 0.2 °C–0.4 °C under low and moderate wind speed conditions, the vertical temperature gradient in this layer can be as much as 200 °C m^−1^–400 °C m^−1^ (ref. 23). Due to the unique condition in the sea surface microlayer, the bacterial composition of this layer or bacterioneuston has been reported to be distinct from that of the underlying water column^6^,^20^,^25^. Bacterioneuston can be affected by solar radiation, ocean wave motions, turbulence, and disruptions of the air-sea interface due to wave breaking. Some bacterioneuston have adapted to the physical conditions within the sea surface microlayer; however, others may have a better chance of survival in the water column below the surface rather than in the sea surface microlayer^26^.

Studying the bacterial content in the sea surface microlayer is an experimental challenge. A number of methods have been developed for surface microlayer sampling over the past decades, including mesh screens^27^, glass plates^28^, and membrane filters^29^. Still, these methods may result in some degree of microbial contamination, either by the mechanical disturbances to the sea surface from the ship hull or by the unintended exposure of the instrument to the underlying water mass.

The focus of our study was to take a snap shot of the bacterial profile of the sea surface, identify the presence of surfactant-associated genera, which may strongly vary in space and time. For this purpose, we have advanced microlayer sampling techniques for the sea surface microlayer, implemented a culture-independent high-throughput sequencing method, and coordinated our field studies with SAR satellite imaging.

## Measurements

In our study, we have implemented the approach described in Franklin *et al*.^25^ to identify the bacterial composition of the sea surface microlayer in open ocean conditions. In Franklin’s study, sampling of surface materials was performed using a 47 mm polycarbonate membrane filter, which was placed on the sea surface with forceps in the proximity of the boat hull. Disruptions of the sea surface microlayer as a result of the ship hull causing wakes may lead to inaccurate microlayer sampling. Therefore, we used a 2.5 m fishing rod to deploy the membrane filters away from the vessel (see Methods).

**Figure 2:**
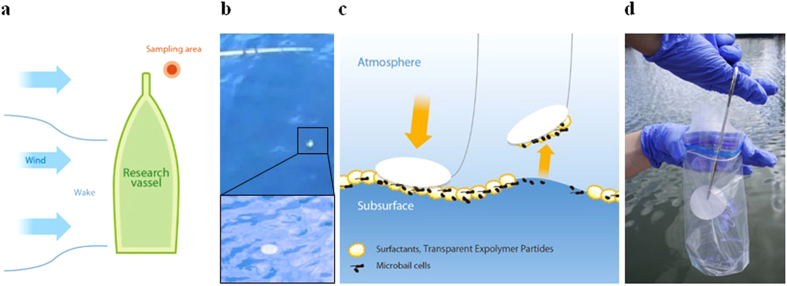
Sea surface microlayer sampling method. **(a**) The sampling is conducted from the bow of the drifting boat, in an area undisturbed by the boat wake. (**b**) A membrane filter is released using a fishing pole to collect samples away from the vessel to avoid contamination from the ship wake. **(c**) Microbial cells in the sea surface microlayer attach to the membrane filter due to surface tension. (**d**) The membrane filter is removed with sterile forceps, stored in a sterile bag and placed on dry ice for laboratory DNA analysis. The sea surface microlayer sampling method was designed to reduce potential contamination of microbial samples. This method included special precautions to avoid hydrodynamic disturbances to the sampling area from the ship hull as well as to identify and eliminate possible microbial contamination during sample collection and handling in the laboratory. The *in situ* sampling method and the subsequent DNA analysis of the bacterial samples are described in more detail in Methods.

The study site was located approximately 8 km east of Port Everglades, Fort Lauderdale, on the Atlantic Coast of Broward County, Florida. The vessel was situated perpendicular to the wind and the membrane filter was released from the bow of a drifting boat away from the disturbances (Fig. 2a). The membrane filter attached to the sea surface primarily due to surface tension and the microbial content was collected (Fig. 2b,c). After approximately 3 to 10 seconds, the filter was retrieved and removed from the nymph hook with sterile forceps. The filter was then stored in a sterile bag where it was immediately placed on dry ice (Fig. 2d). Subsurface microbial samples were taken from 0.2 m depth in an area undisturbed by the ship using tubing and a peristaltic water pump (see Methods).

A total of over 100 *in situ* samples were taken during experimentation in the Straits of Florida over several days in 2010 and 2011. All stages of sampling were recorded on video in order to identify and discard unsuccessful sampling attempts. Samples taken on July 10, 2010 were chosen for the analysis based on satellite overpass, no rain conditions, and the presence of a pronounced slick area in the Straits of Florida. During this day, single samples from each location, sea surface microlayer slick and non-slick area, and subsurface water corresponding to the slick and non-slick areas, were taken. Samples taken on September 10, 2011 were included in the analysis in order to evaluate contamination issues during sample handling in the field and laboratory analysis. During this day, we took only control and non-slick samples (no slicks were observed). The control samples included one filter exposed to the air and one prepared along with other filters but never exposed to the environment. The non-slick samples included one filter from the sea surface microlayer and one from the water column. All filters were processed in the same manner throughout the course of the laboratory work using 454 high-throughput sequencing, and bioinformatics analysis (see Dataset 1 in Supplementary Information).

## Results

**Figure 3:**
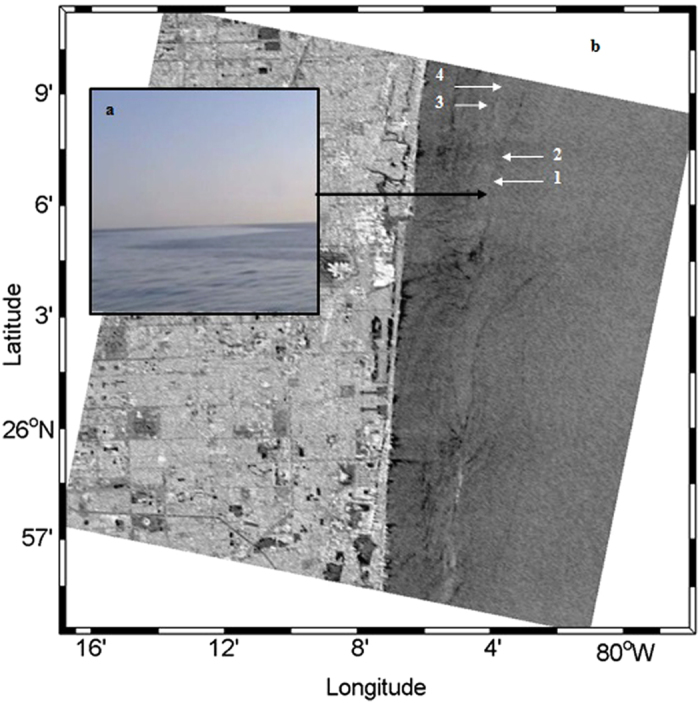
Case study on July 10, 2010 in the Straits of Florida. (**a**) Photographic image of a slick. The glossy area represents the slick. (**b)** SAR image of the sampling areas captured with the RADARSAT-2 satellite at 11:23 UTC on July 10, 2010. The image is in C-band VV-polarization. The corresponding operational beam mode is fine quad-polarization mode. The incidence angle is between 26.9 (near) –28.7° (far) and the resolutions in range and azimuth directions are 5.4 and 8.0 m. A 3*3 Lee Filter was applied to reduce the speckle existing on the SAR image. The radiometric error for RADARSAT-2 fine quad-polarization imaging model is smaller than 1 dB. Slicks appear in the respective SAR image as dark lines. The study site was located approximately 8 km off the Atlantic Coast of Fort Lauderdale, Florida; wind speed was around 1.8–2.6 ms^−1^; wind direction, 240–260^o^. 1 – Slick sampling, sea surface microlayer. 2 – Slick sampling, subsurface. 3 – Non-slick sampling, sea surface microlayer. 4 – Non-slick sampling, subsurface.

In the case study on July 10, 2010 (Fig. 3), the *in situ* bacterial sampling was coordinated with a RADARSAT 2 satellite overpass. Slicks were visually identified based on their glossy appearance (Fig. 3a), which provided guidance in collecting *in situ* samples exactly within the slick area. Slicks appeared in the respective SAR image as dark lines (Fig. 3b). Sampling locations in the slick and non-slick area are indicated in the SAR image shown in Fig. 3b.

In this case study, the 454 sequencing analysis was used to get the first insight into the overall bacterial composition of the sea surface microlayer and water column in the slick and non-slick areas. The results of the 454 sequencing analysis of samples were organized by operational taxonomic units (OTUs—see Methods) and subsampled to 382 reads in order to match the lowest sequence number obtained for the slick sea surface microlayer 2010 sample using QIIME 1.8.0^30^ (see Dataset 1 in Supplementary Information). The OTUs found on control filters are treated as an indication of potential contamination during sample handling at sea or in laboratory.

**Figure 4:**
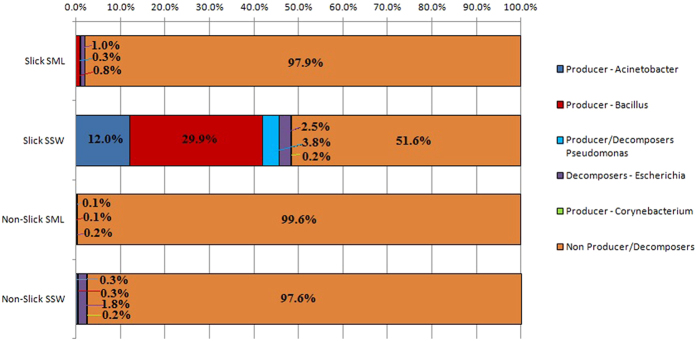
Relative abundance of surfactant-associated genera observed during the experiment shown in Fig. 3. Genus-level taxonomic assignments of 16S rRNA gene were used to determine relative abundance of the potential surfactant producers and decomposers, and non-producer/decomposers in each location (SML and SSW stand for sea surface microlayer and subsurface water, respectively).

The following genera of surfactant-associated bacteria known from literature (see e.g. ref. 15)have been identified from the 454 sequencing analysis: potential surfactant producers *Acinetobacter* spp., *Bacillus* spp., *Corynebacterium* spp., and *Pseudomonas* spp. (also a degrader), and a surfactant degrader *Escherichia* spp. Most intriguingly, the greatest abundance of potential surfactant producers, *Acinetobacter* spp. and *Bacillus* spp., was detected in the subsurface below the sea slick rather than in the sea surface microlayer sample (Fig. 4).

Analysis of the 2010 data showed indication of possible contamination for some bacteria genera during sample handling and lab analysis. In order to address this issue, control filters were introduced in the analysis. A related test was conducted during the 2011 field campaign and certain OTUs, belonging to the *Acinetobacter, Corynebacterium,* and *Eschierichia* genera, were found on control filters (see Methods and Dataset 1 in Supplementary Information), which were an indication that there was higher probability of contamination with these genera during field and lab handling of samples. There was no contamination detected for *Bacillus,* and *Pseudomonas* (no OTUs belonging to these genera have been found on control filters).

## Discussion

The implementation of the new sampling techniques and analysis has allowed us to observe the differences between the compositions of surfactant-associated bacteria in the slick and non-slick areas, and also between the sea surface and subsurface water. The surfactant producers, *Acinetobacter* spp. and *Bacillus* spp. appear to be present almost exclusively in the slick area and mostly localized in the water column rather than in the sea surface microlayer (Fig. 4). (Note that *Acinetobacter* spp. may be subject to contamination during sample handling in the field or laboratory). This observation indicates that production of surfactants may take place in the organic-rich water column below the sea surface. Surfactants produced by the marine bacteria can then be transported to the sea surface by physical processes including diffusion, convection, advection, and bubble scavenging. The organic materials on the sea surface, as well as those dissolved in the water column, can potentially form a slick on the sea surface within a certain range of wind-wave conditions.

Our taxonomic analysis based on 16S rRNA marker genes gives us insight into the surfactant-associated bacteria and their potential surfactant related activities in the near-surface layer of the ocean. There is insufficient information on the functional traits of marine bacteria related to production of surfactants, because a significant amount of surfactant producing bacteria in the ocean has not yet been identified. An approach including comparative genomics and transcriptomics analysis^31^ can be involved in the future when sufficient amount of surfactant associated genes of marine bacteria will be known.

Homology searches indicated that one of the highest number of OTU, denovo1229 (see Methods and Dataset 1 in Supplementary Information), was 99% identical to *Bacillus litoralis* found in the tidal flat of the Yellow Sea, Korea^3^. It is unknown if *B. litoralis* is directly involved in hydrocarbon biodegradation through production of surfactants. We can speculate that it is involved in biodegradation, since this species was isolated from the same hydrocarbon environment as the other known exopolysaccharide producers *B. cereus*, *B. subtilis*, and *Pseudomonas stutzeri*^33^,^34^,^35^. The latter are also known for the biodegradation of hydrocarbons^36^,^37^. There is no study showing that *B. litoralis* was associated with oil samples from the Deepwater Horizon oil spill disaster, which occurred on April 20, 2010 and was capped on 15 July 2010. Therefore, we cannot eliminate the possibility that dissolved oil was present in the water column during our sampling on July 10, 2010 in the Straits of Florida.

Our 16S rRNA amplicon sequencing approach helped us identify potential surfactant-associated genera in the sea surface microlayer as well as in the near-surface layer of the ocean. However, only a few surfactant producer and degrader species are known from the literature, which lets us speculate that there is a larger number of surfactant-associated taxa yet to be identified in the near-surface layer of the ocean. The reasonable next steps would be to collect and amplify DNA in a manner that quantitatively and statistically reflects the microbial community. Notably, in order to gain a more comprehensive composition and distribution of surfactant associated bacteria and surfactants on the sea surface, it is necessary to collect a larger number of samples in a wider range of environmental conditions. A companion paper^38^ implementing the method developed in this study includes analysis of larger number of samples but only for one bacteria genera (*Bacillus*).

The sampling in our study was performed under low wind speed conditions; however, the obtained results may provide an insight into a wider range of environmental conditions, since the surfactant-producing bacteria, if predominantly located in the water column below the wave-stirred layer, might be less affected by adverse weather conditions. Under high wind speed conditions, the near-surface layer of the ocean is saturated with air-bubbles due to wave breaking, while the rising air-bubbles scavenge surfactants in the water column. Due to alteration of surface tension by surfactants, the air-bubbles affect air-sea gas exchange, including greenhouse gases^39^, and also the bubble-bursting mechanism, which is an important factor in the production of climate-active marine aerosols^3^,^4^,^39^. We thus speculate that surfactant-producing and degrading bacteria may represent a factor in climate and climate change, alongside phytoplankton and other contributors to the surfactant pool.

Slicks on the sea surface were typically observed under low and moderate wind speed conditions. Under these conditions, the SAR backscatter was dominated by surface (Bragg^22^) scattering by short gravity-capillary waves. If surfactants are present, they tend to dampen the capillary waves, and thus they can be detected in SAR imagery. Under high winds, the air-sea interface is disrupted by bubble-bursting and covered by sea spray and foam, essentially tending to form a two-phase environment. In these conditions, the SAR backscatter is dominated by volume-scattering, rather than surface Bragg-scattering by short gravity-capillary waves. While Bragg-scattering is known to saturate for increasingly high winds, in the sense that the backscatter signal reaches a limiting plateau and then begins to decrease, saturation does not appear to occur for the volume-scattering, which can be measured by satellite cross-polarization channels^22^. In this latter high wind scenario, bubbles, sea spray and foam apparently depend on surface tension effects. As a result, polarimetric SAR imagery can potentially be affected by the presence of surfactants, and thus surfactant-associated bacteria in the water column to some extent, even in high wind speed conditions.

Unlike coloured algal blooms, surfactant-associated bacteria may not be visible in ocean colour imagery. Having the ability to detect these “invisible” surfactant-associated bacteria using SAR has immense benefits in all-weather conditions, regardless of cloud, fog, or daylight. This is particularly important in very high winds, because these are the conditions when the most intense air-sea gas exchanges and marine aerosol production take place. Therefore, in addition to colour satellite imagery, SAR satellite imagery may provide additional insights into a global picture of biophysical processes at the boundary between the ocean and atmosphere, air-sea greenhouse gas exchanges, and production of climate-active marine aerosols.

Here we have focused on the role of bacteria in the process of surfactant production and degradation in the marine environment. This is a pilot type study, with inherent limitations. The main limitations included the small sample size and small size of the filter used for DNA collection. For future studies, a more comprehensive approach should be implemented, including community-level analysis (metagenomics).

## Methods

### Sampling Methods

The surface sampling method consisted of attaching a polycarbonate membrane filter (47 mm diameter) to a fly-fishing nymph hook, which was pre-sterilized (Fig. S1, Supplementary Information). The fly-fishing nymph hook was then tied to a 0.2 m piece of sterilized fly-fishing line with a loop at the opposite end. (The membrane filter, hook, and line were all placed inside a sterile, plastic zip-lock bag until sampling commenced.)

The small size of the filter in a diameter of 47 mm made our sample collection easier in terms of handling and applying aseptic sampling techniques. However, the low biomass filter sample yielded a low concentration of genomic DNA, which required a relatively large number of PCR cycles in compensation. The related PCR errors, such as chimeric sequences, were discarded during the bioinformatic analysis in this study. Yet, such a removal of erroneous sequences may cause a large loss of overall sequence reads and lead to shallower sequencing depth. The alpha diversity plots demonstrated that the nearly leveled off accumulation curve of the slick subsurface water sample (Fig. S2, Supplementary Information) was well censused as compared to the slick sea surface microlayer sample. The steeper initial curve of this slick microlayer sample implied a higher species diversity present in the sea slick, although the low number of overall reads suggested that an increased sampling effort might result in a more accurate estimation of species diversity. Nonetheless, this study focused on the comparison of specific OTUs belonging to *Bacillus* spp. and *Acinetobacter* spp., within the rarefied samples of 382 reads. This initial portion of the curves up to 382 reads represent the relatively dominated species as rarefaction curves generally raise rapidly at beginning, sampling the most common species, but curves begin to level off while sampling rarer species.

Subsurface microbial samples were taken from 0.2 m in depth in an area undisturbed by the ship. A portable environmental water sampler (Barnant Co., Barrington, IL) was used to collect subsurface water. The water sampler has a rotor that generates a peristaltic motion. A flexible tube is inserted into the rotor that continuously pumps the fluid through the flexible tubing, which eliminates contact between the pump and fluid. To avoid cross contamination, new tubing was used to collect each water sample and sterilized prior to use. To facilitate the collection of water samples away from the vessel wake, the tubing was inserted in a 1m long PVC pipe and held steady on the downwind side of the drifting boat. The tubing was marked 0.2 m away from the suction end in order to ensure accurate sample depths. The pump was started once the suction end was submerged to the correct depth and pumped for a few minutes in order to remove possible residual contamination inside of the plastic tubing. Approximately 500 ml of water sample was then collected into a sterile bag. Immediately afterward, several membrane filters were soaked in the collected water for 3 to 10 seconds. Each membrane filter was then placed in a sterile bag and stored on dry ice. These subsurface sampling techniques are implemented to replicate, as close as possible, the sea surface microlayer filter collection procedure.

### Genomic DNA extraction from filter membranes

Genomic DNA was extracted directly from each membrane filter sample in sterile laboratory conditions by utilizing the QIAamp DNA Investigator Kit (QIAGEN Inc., Valencia, CA). Each filter was cut into small pieces and added to individual 1.5 ml tubes. We followed the merchant instructions for the remainder of the procedure. NanoDrop1000 spectrophotometer (Thermo Scientific, Wilmington, DE) was used to measure concentration and purity of the samples.

### 454 GS FLX library preparation

The 16S rRNA genes of approximately 1100 bp in length were amplified with universal 16S rRNA primers 27F (5′-AGAGTTTGATCMTGG-3′) and 1492R (5′-TACCTTGTTACGACTT-3′)^40^. The PCR cycle setting consisted of an initial heating at 95 °C for 2 min, followed by 30 cycles of, 95 °C for 30 sec, 45 °C for 30 sec and 72 °C for 1 min, and a final elongation step at 72 °C for 5 min. After the 30 cycle amplification, there were no bands observed on an agarose gel (1.5%) except a positive control, which was probably due to the limited genomic DNA material available on filters.

The PCR products diluted by a factor of 10,000 were used to perform nested fusion PCR. Barcoded fusion primers were designed using the universal 16S rRNA primers 357F (5′-TACGGGAGGCAGCAG-3′) and 805R (5′-GACTACCAGGGTATCTAATC-3′)^41^, which cover the V3 and V4 hypervariable regions (average 438 bp in length). With the designed primer sets, the nested fusion PCR amplification was performed using the FastStart High Fidelity PCR System (Roche Diagnostic Corp., Indianapolis, IN). The same cycle settings were used as indicated above. The samples were size selected using the QIAquick Gel Extraction Kit, and purified using the QIAamp DNA Investigator kit (QIAGEN Inc., Valencia, CA). Emulsion PCR and unidirectional 454 GS-FLX sequencing were performed at the Interdisciplinary Center for Biotechnology Research, Genomics Division, at the University of Florida.

### Bioinformatics and taxonomic analyses

From a total of 88,198 raw sequences of 8 samples collected on 10 July, 2010 and 10 September, 2011, we obtained 23,778 quality-filtered, non-chimeric sequences. Of those filtered sequences, 10, 593 were used in Fig. 4.

Sequences were filtered out during the demultiplexing step, if they had >6 ambiguous base calls, minimum average quality score <25, minimum sequence length <200nt, maximum length of homopolymer >6, and contain any primer mismatches. The demultiplexed sequences were denoised using QIIME denoiser^42^. Operational Taxonomic Units (OTUs) were picked according to their sequence similarity to 97% with UCLUST algorithm^43^. PyNAST^44^ was then used to align the OTUs with an alignment template obtained from the Greengenes 16S rRNA gene database^45^. Chimeric sequences were removed using ChimeraSlayer^46^ before taxonomy was assigned with the Ribosomal Database Project (RDP) classifier^47^. OTU tables were generated and rarefied to 382 reads in order to match the lowest sequence number obtained for the slick sea surface microlayer (Slick SML) 2010 sample (Fig. S2b and Dataset 1 in Supplementary Information). Alpha diversity rarefaction plots were created in QIIME using Observed Species metrics (Fig. S2, Supplementary Information). The rarefied OTU table was used to determine the relative abundance of surfactant-associated genera (Fig. 4). The raw sequence reads have been deposited in the NCBI Sequence Read Archive database under accession number PRJNA280411.

In a separate project^38^, the samples taken on July 10, 2010 have also been analyzed using Real time PCR for genus *Bacillus* and shown consistent results with our sequencing described above (Fig. S3, Supplementary Information).

## Additional Information

**How to cite this article**: Kurata, N. *et al.* Surfactant-associated bacteria in the near-surface layer of the ocean. *Sci. Rep.* **6**, 19123; doi: 10.1038/srep19123 (2016).

## Supplementary Material

Supplementary Information

Supplementary Dataset 1

## Figures and Tables

**Figure 1:**
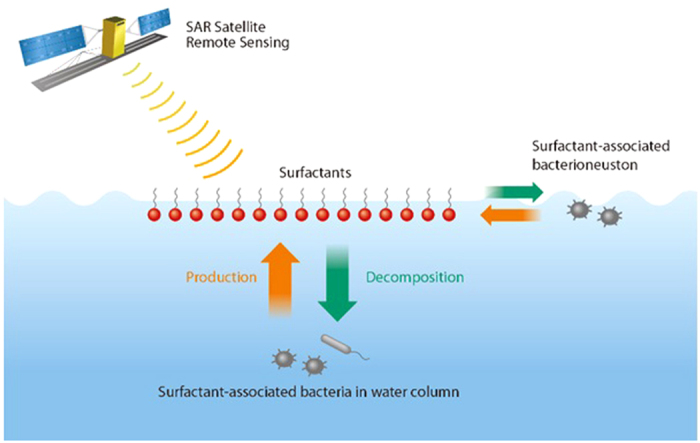
Schematics of the experiment to reveal the link between surfactant-associated bacteria, sea slicks, and synthetic aperture radar satellite remote sensing. Surfactants are capable of dampening the short capillary ocean surface waves and smoothing the sea surface^18^,^27^. SAR can detect areas with concentrated surfactants or sea slicks, which appear as dark areas on the SAR images.
