# Supplementary material for: Surfactant-associated bacteria in the near-surface layer of the ocean
Source: Sci Rep. 2016 Jan 12;6:19123. doi: 10.1038/srep19123 (PMC4709576; doi:10.1038/srep19123)

**Surfactant-associated bacteria in the near-surface layer of the ocean: Supplementary information**

**Naoko Kurata^1*^, Kate Vella^1^, Bryan Hamilton^1^, Mahmood Shivji^1^, Alexander Soloviev^1,2*^, Silvia Matt^3^, Aurelien Tartar^4^, William Perrie^5^**

^1^ Oceanographic Center, Nova Southeastern University, Dania Beach, FL, USA

^2^ Rosenstiel School of Marine and Atmospheric Science, University of Miami, Miami, FL, USA

^3^ Naval Research Laboratory, Stennis Space Center, MS, USA

^4^ Division of Math, Science and Technology, Nova Southeastern University, Fort Lauderdale, FL, USA

^5^ Fisheries and Oceans Canada, Bedford Institute of Oceanography, Nova Scotia, Canada

* Correspondence and requests for materials should be addressed to N.K. (email: kurata.naoko.jp@gmail.com) or A.S. (email: soloviev@nova.edu)

**Figure S1 |** Equipment preparation: the fishing line and hook are sterilized and carefully stored in a sterile plastic bag until use to minimize contamination.

**a b**


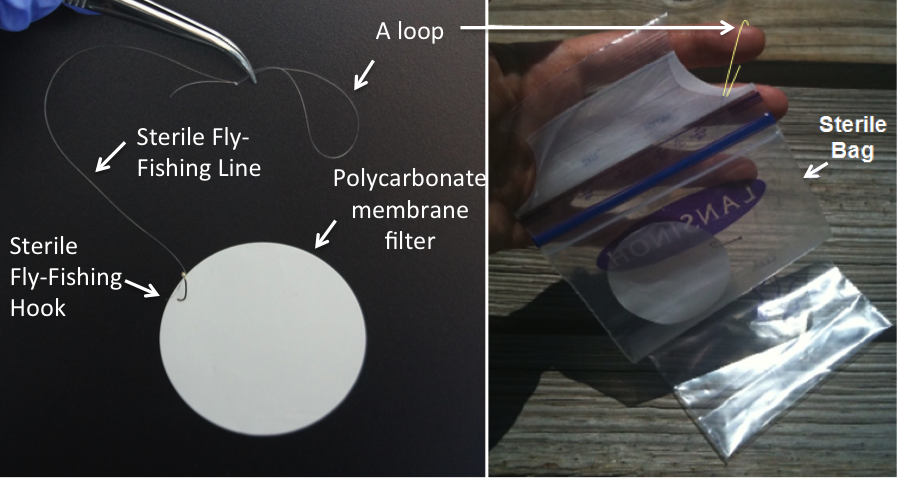


**Figure S2** | Alpha diversity of the identified 16S rRNA OTUs. **a**. The sample depth of four filter samples collected during the 2010 case study. **b**. A subset of plot a (rarefied to 382 reads). The four filter samples presented here are the slick sea surface microlayer (SML), slick subsurface water (SSW), non-slick SML, and non-slick SSW.

**a b**


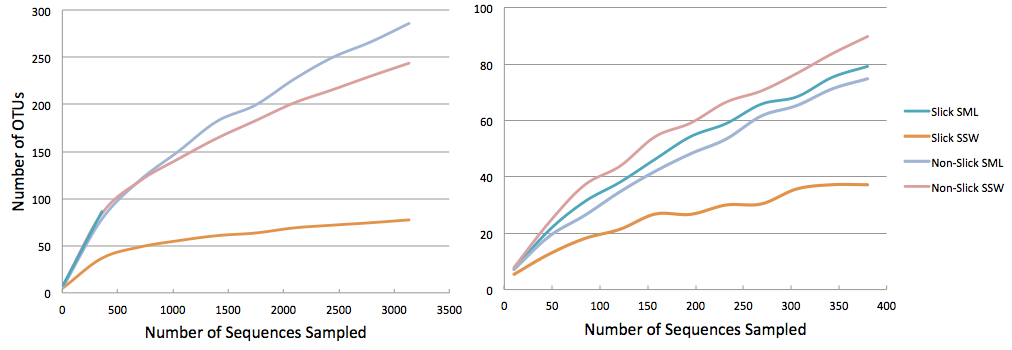


**Figure S3**| Abundance levels of the surfactant associated genus *Bacillus* from samples collected during the 2010 case study in the Straits of Florida. **a.** Results using 454 sequencing techniques. The abundance of *Bacillus*genus identified on each sample relative to all bacterial genera; **b.** Results using real time PCR techniques. Real time PCR relative abundance is given in technical units equal to 10^7^/2*^ct^*, where *ct* is the cycle count when the amount of fluorescence reaches a determined threshold level for each sample during a quantitative PCR run.

**a b**


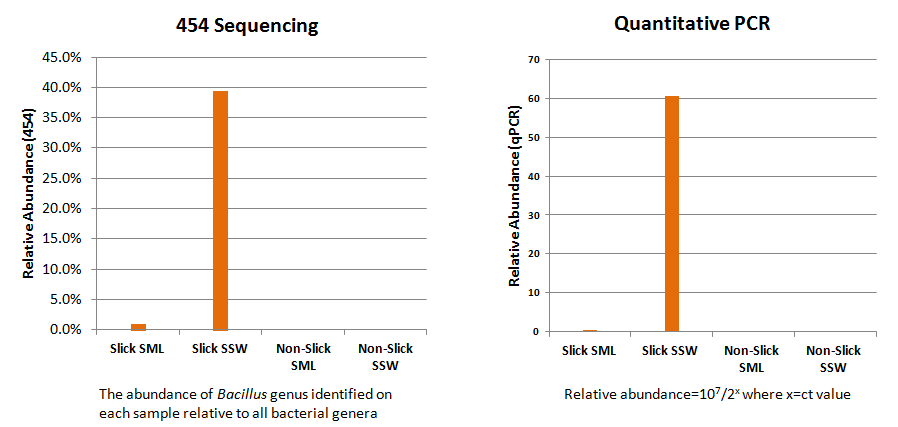

Supplement: Supplementary Information [file srep19123-s1.doc]
